# CT after Lung Microwave Ablation: Normal Findings and Evolution Patterns of Treated Lesions

**DOI:** 10.3390/tomography8020051

**Published:** 2022-03-01

**Authors:** Valentina Vespro, Maria Chiara Bonanno, Maria Carmela Andrisani, Anna Maria Ierardi, Alice Phillips, Davide Tosi, Paolo Mendogni, Sara Franzi, Gianpaolo Carrafiello

**Affiliations:** 1 Department of Radiology, IRCCS Foundation Ca’ Granda Ospedale Maggiore Policlinico, 20122 Milan, Italy; valentina.vespro@policlinico.mi.it (V.V.); mariachiara.bonanno@unimi.it (M.C.B.); maria.andrisani@policlinico.mi.it (M.C.A.); annamaria.ierardi@policlinico.mi.it (A.M.I.); alice.phillips@unimi.it (A.P.); gianpaolo.carrafiello@unimi.it (G.C.); 2 Department of Health Sciences, University of Milan, 20122 Milan, Italy; 3 Thoracic Surgery and Lung Transplantation Unit, IRCCS Foundation Ca’ Granda Ospedale Maggiore Policlinico, 20122 Milan, Italy; davide.tosi@policlinico.mi.it (D.T.); paolo.mendogni@policlinico.mi.it (P.M.)

**Keywords:** lung cancer, percutaneous treatments, microwave ablation, computed tomography, CT

## Abstract

Imaging-guided percutaneous ablative treatments, such as radiofrequency ablation (RFA), cryoablation and microwave ablation (MWA), have been developed for the treatment of unresectable primary and secondary lung tumors in patients with advanced-stage disease or comorbidities contraindicating surgery. Among these therapies, MWA has recently shown promising results in the treatment of pulmonary neoplasms. The potential advantages of MWA over RFA include faster ablation times, higher intra-tumoral temperatures, larger ablation zones and lower susceptibility to the heat sink effect, resulting in greater efficacy in proximity to vascular structures. Despite encouraging results supporting its efficacy, there is a relative paucity of data in the literature regarding the role of computer tomography (CT) to monitor MWA-treated lesions, and the CT appearance of their morphologic evolution and complications. For both interventional and non-interventional radiologists, it is crucial to be familiar with the CT features of such treated lesions in order to detect incomplete therapy or recurrent disease at early stage, as well as to recognize initial signs of complications. The aim of this pictorial essay is to describe the typical CT features during follow-up of lung lesions treated with percutaneous MWA and how to interpret and differentiate them from other radiological findings, such as recurrence and complications, that are commonly encountered in this setting.

## 1. Introduction

The treatment of lung tumors, both primary and metastatic, has undergone substantial diversification in the last two decades [1,2,3,4,5]. Although surgery, consisting of pneumonectomy or lobectomy with lymph node sampling, remains the gold standard treatment, only 15% of patients present a resectable lesion at diagnosis and many of them are not eligible for surgery because of poor cardiopulmonary reserve, unfavorable tumor localization or systemic metastatic disease [6]. For patients who are not surgical candidates, new, minimally invasive treatment options, including percutaneous thermal ablation therapies and high-dose radiation therapies, have emerged as safe and effective treatment alternatives [7,8].

Among the different ablation therapies, microwave ablation (MWA) is a relatively new technique, whose effectiveness and safety were demonstrated for the first time in a large study of 50 patients with lung cancer in 2008 by Wolf et al. [7]. MWA can be applied to different types of tumors and has recently shown promising results, especially in the treatment of pulmonary neoplasms [9]. MWA offers all the benefits of radio frequency ablation (RFA), as well as some substantial advantages, such as larger ablation volumes with a larger active heating zone, reduced procedure time, less intraprocedural pain and higher efficacy on lesions in proximity to vascular structures [10]. The latter is explained by the less susceptibility of MWA to the cooling effect of flowing blood, a phenomenon known as the ‘heat-sink effect’, which limits the effectiveness of ablation on lesions in proximity to vessels larger than 3 mm [11,12].

However, there are also several challenges to consider while performing this new technique. A major one is the post-procedural assessment of adequate response to treatment. Since the ablated tumor and the surrounding ablated lung tissue remain in place during MWA, the direct histopathologic verification of complete tumor ablation is not possible [13]. In this scenario, post-procedural cross-sectional imaging represents the most reliable and feasible means of evaluating and ensuring treatment success, both immediately after the procedure and during follow-up. A comprehensive understanding of the evolution of the MWA site on imaging is, therefore, essential for both interventional and non-interventional radiologists, in order to promptly detect incomplete treatment or recurrent disease at an early stage.

Computed tomography (CT), which also allows detection of procedure-related complications, metachronous tumors and metastatic disease, is primarily used for this purpose [10]. However, there is a relative paucity of data in the literature regarding the role of CT to accurately monitor MWA-treated lesions and their morphologic evolution during follow-up. Moreover, despite the well-established role of CT in this scenario, there is currently no unanimous consensus regarding the best imaging modality or the optimal timing to assess success or failure of treatment [10]. The purpose of this pictorial essay is to describe a potential timing for post-MWA imaging surveillance using CT by reviewing the expected and typical radiological appearance of post-ablation lung lesions during a predetermined follow-up (1–3–6 months), in order to differentiate them from radiological signs of recurrence or complications.

## 2. Imaging Follow-Up

At our institution, contrast-enhanced CT is the imaging modality of choice in order to plan the treatment and the following surveillance, while combined Positron Emission Tomography (PET)/CT may be considered an option at 6 months in doubtful cases. Cone-beam CT (CBCT) is used for intraprocedural targeting, monitoring and assessment of immediate post-procedural response and complication.

For follow-up examination, a multi-detector row helical 64-slice CT scanner (SOMATOM Definition—Siemens Healthcare) is used. Thoracic nonenhanced and contrast material-enhanced CT images are acquired with 3- and 1-mm collimation. The contrast-enhanced scans are acquired 60 s after injection of iodinated contrast agent (Iopamiro 370, Bracco Healthcare; 1.35 mg/kg body weight) at an injection rate of 3 mL/s, followed by injection of 40 mL saline at a rate of 3 mL/s.

The first CT examination is usually performed 1 month after MWA, in order to confirm complete treatment and establish a reference imaging for subsequent follow-up examinations, which are performed at 3- and 6-month intervals, in conformity with protocols adopted in most centers, with the only difference that no CT is performed after 24 h, as the CBCT is considered sufficient for immediate post-procedural examination [7,8,14].

## 3. Normal Imaging Features

### 3.1. Post-Procedural Cone-Beam CT

**Figure 1:**
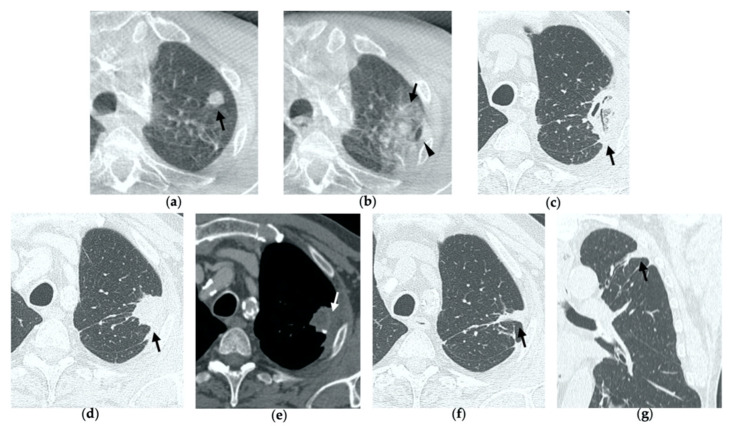
74-year-old man with a pulmonary metastasis from bladder urothelial carcinoma. (**a**) Cone-beam CT image of the left upper lobe metastasis (black arrow) prior MWA. (**b**) Cone-beam CT image obtained post-procedure shows hazy GGO of the ablation site surrounding the treated nodule (black arrow) and a small layer of lateral pneumothorax (arrowhead). (**c**) Axial 1-month follow-up CT image shows a large consolidation with inner cavitation (black arrow). (**d**,**e**) Axial 3-month follow-up CT image shows resolution of the cavitation and decrease in size of the consolidation (black arrow) (**d**) and demonstrates peripheral mild enhancement with no central contrast material uptake (white arrow) (**e**–**g**). Axial (**f**) and coronal (**g**) CT images obtained after 10 months show a residual fibrotic band (black arrow).

**Figure 2:**
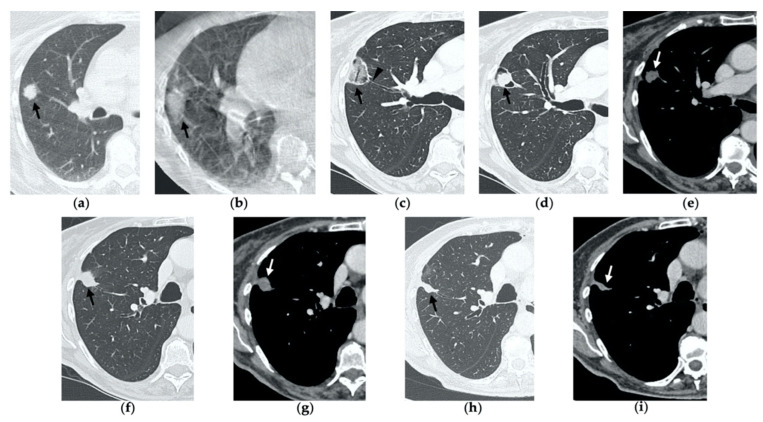
69-year-old woman with a biopsy-proved NSCLC in the right upper lobe. (**a**) Pre-treatment axial CT. (**b**) Cone-beam CT image post-MWA shows GGO (black arrow) among the ablation site. (**c**–**e**) Axial 1-month follow-up CT shows (**c**) a large consolidation with well-defined margins, inner hypoattenuating bubbles and a cavitation (black arrow) in communication with a peripheral bronchus (arrowhead); (**d**) on a different level, central necrotic material is seen within the cavitation (black arrow) (**e**) with no contrast enhancement (white arrow). (**f**,**g**) Axial 6-month follow-up CT images show resolution of the cavitation and decrease in size of the consolidation (black arrow) with no contrast material uptake (white arrow). (**h**,**i**) Axial 9-month follow-up CT image shows a linear fibrotic band with no contrast enhancement.

**Figure 3:**
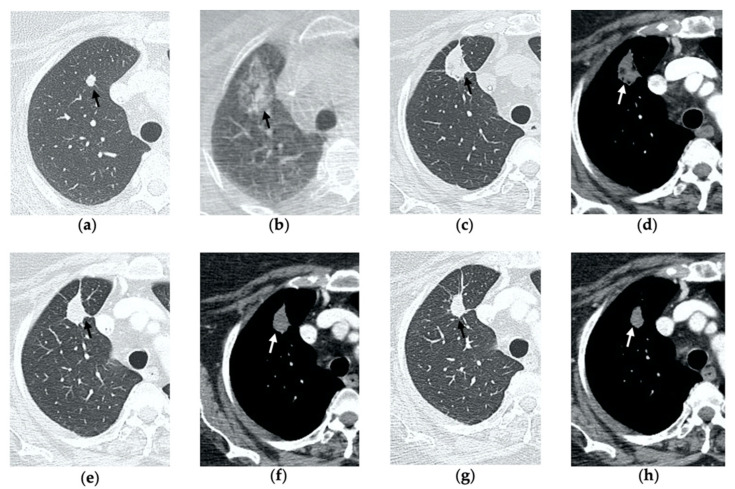
63-year-old woman with pulmonary metastasis from colorectal cancer (CRC) in the right upper lobe. (**a**) Axial CT of the metastasis (black arrow) prior to treatment. (**b**) Cone-beam CT image obtained post-procedure shows hazy GGO (black arrow) of the ablation site surrounding the treated nodule. (**c**,**d**) Axial 2-month follow-up CT images show a large consolidation with hypoattenuating bubbles (black arrow) and no contrast uptake (white arrow). (**e**,**f**) Axial 5-month follow-up CT images demonstrate a decrease in the size of the consolidation (black arrow) with no central contrast enhancement (white arrow). (**g**,**h**) Axial CT images obtained after 8 months show minimal further decrease in size with no signs of residual disease.

**Figure 4:**
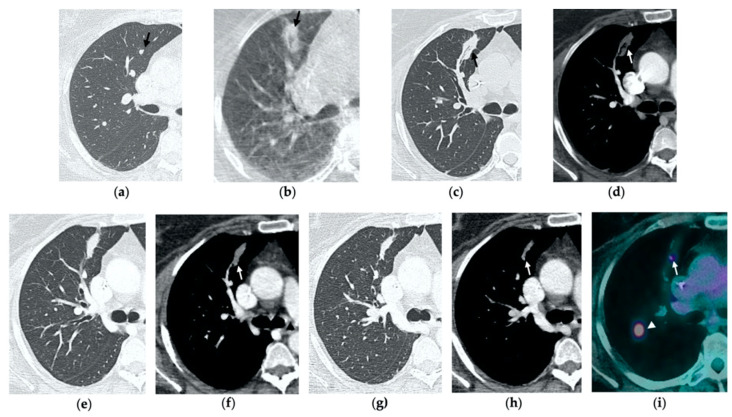
63-year-old-woman (same patient in Figure 3) with pulmonary metastasis from CRC in the right upper lobe. (**a**) Axial CT before treatment (black arrow). (**b**) Cone-beam CT image obtained at the end of the procedure shows GGO (black arrow) around the treated lesion. (**c**,**d**) Axial 2-month follow-up CT images show an elongated consolidation with hypoattenuating bubbles (black arrow) and no contrast uptake (white arrow). (**e**,**f**) Axial 5-month follow-up CT images demonstrate a tiny nodular uptake of contrast on the posterior margin of the consolidation (white arrow), suggestive of residual disease. (**g**,**h**) On axial CT images after 8 months the nodular enhancement persists (white arrow). (**i**) PET/CT image at 9 months proves residual disease on the treated lesion (white arrow) as well as simultaneous metastasis (white arrowhead) in the posterior segment.

**Figure 5:**
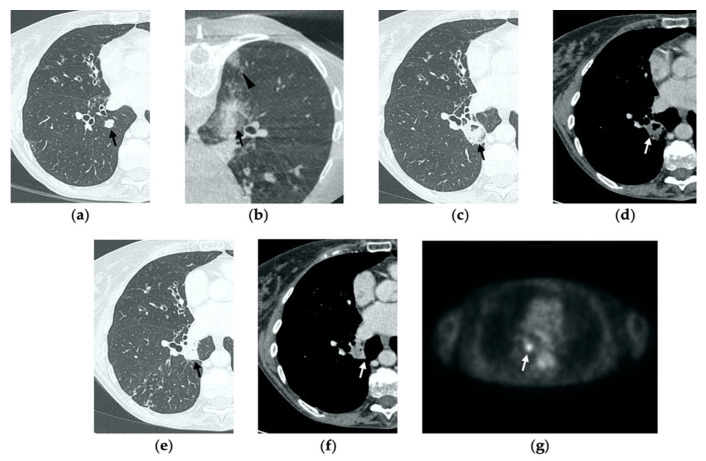
77-year-old-woman with pulmonary metastasis from CRC in the right lower lobe. (**a**) Axial CT images prior to treatment (black arrow). (**b**) Prone cone-beam CT image post-MWA shows GGO of the ablation site (black arrow) and alveolar hemorrhage along the needle tract (arrowhead). (**c**,**d**) Axial 1-month follow-up CT shows a subpleural consolidation with well-defined margins, inner hypoattenuating bubbles, a central cavitation (black arrow) and no contrast enhancement (white arrow). (**e**,**f**) Axial 6-month follow-up CT images demonstrate resolution of the cavitation, decrease in size of the consolidation (black arrow) and focal uptake of contrast material (white arrow) consistent with a diagnosis of residual disease. (**g**) The diagnosis is confirmed by PET/CT performed at 7 months (white arrow).

On CBCT images performed immediately after MWA, the ablation site appears as a well-demarcated oval or wedged-shaped area of hazy ground glass opacification (GGO) surrounding the treated lesion (Figure 1b, Figure 2b, Figure 3b and Figure 4b) [15]. The GGO zone resembles a foreseen response as it corresponds to local hyperemia, congestion, hemorrhage, inflammation and necrosis induced by the thermal ablation effect [16,17,18]. As a result, the overall ablation zone appears larger than the original tumor since it represents both the tumor and the perilesional opacity [8,15]. Since at this stage it is not possible to assess the complete radicality of the ablation, the treatment is usually considered optimal if the lesion is completely surrounded by the GGO zone. An irregular longitudinal sub-solid area of parenchymal hemorrhage may be observed along the needle tract or in proximity to the ablation site (Figure 5b) [8,19]. Post-procedural examination also allows the detection of early complications, such as pneumothorax, which should be strictly monitored through chest radiographs in the hours immediately after MWA, as well as the following days [20].

### 3.2. Contrast-Enhanced CT at 1 Month

By the first month after MWA, the rim of parenchymal GGO has dissolved in most patients as a result of regressing parenchymal edema, inflammation and hemorrhage, and the ablation site appears as an area of consolidation with a mean diameter still larger than the preablation zone (Figure 3c,d and Figure 4c,d) [21]. It is, therefore, crucial in this phase to measure the area of consolidation by its maximum axial diameter in order to thoroughly compare it during the following phases [7]. The consolidation may demonstrate inner cavitation or a central hypoattenuating area with reduction in contrast material uptake, along with a mild peripheral enhancement layer as an expression of reactive hyperemia, which should present smooth with linear margins (Figure 1c) [7,15]. This phenomenon must be referred to benign periablational enhancement and it should be differentiated from pathological contrast uptake, which is usually more irregular and nodular-shaped [7]. It is, therefore, pivotal to perform CT before and after contrast material administration in order to adequately evaluate the enhancement features of the treated tumor. The ablation site may also show hypoattenuating bubbles or a cavity with thin walls, containing solid tissue with reduced contrast enhancement, necrotic material or air-fluid levels, and a communication between the cavitation and a bronchus may be recognized (Figure 2c–e) [7,16]. The latter being a common finding since the necrotic tissue may be evacuated through a bronchus and it should not be mistaken for rare although possible complications, such as an abscess or a broncho-pleural fistula (BPF). Unlike the normal cavitary changes of the ablation area, an abscess is a rare complication (0.5%) [20] and appears as a cavity with thick walls, irregular internal contours and air-fluid level, and must be suspected when fever and laboratory signs of infection are present [22]. Pleural changes are also common findings, especially in peripheral lesions, including pleural thickening in the region of pleura traversed by the microwave antenna, pleural retraction and effusion [15]. Reactive mediastinal lymphadenopathy often occurs at an early stage, and it should not be considered a sign of tumor progression [7,23].

### 3.3. Contrast-Enhanced CT at 3 Months

On CT images obtained at 3-month follow-up, the size of the ablation zone should be the same or still larger than the baseline tumor, although it undergoes further involution compared to the early phase, as during the fibrosis process the wall thickness and the previously depicted cavities progressively decrease (Figure 1d) [7]. The attenuation of the ablated tissue decreases, as there is no more central contrast material uptake in relation to the local necrotic changes, while the peripheral benign enhancement may persist or decrease (Figure 1e). Overall, the size of the ablation area at this stage should become stable along with a decrease in wall thickness [7,8].

### 3.4. Contrast-Enhanced CT at 6 Months

After 6 months, the ablation site undergoes further involution and there should not be any inner contrast enhancement, except for the persistent benign periablational area (Figure 2f,g) [7]. The previously mentioned cavities decrease in size and may completely disappear. CT images may show fibrotic scarring without contrast enhancement and mild architectural parenchymal distortion of the surrounding lung (Figure 1f,g) [24]. At this stage, small treated nodules may already show a linear fibrotic evolution on CT images (Figure 2h,i).

## 4. Residual or Recurrent Disease

During follow-up, there are several evolution patterns of the ablation site, which should promptly raise the suspicion of incomplete ablation or local recurrence.

On post-procedural CBCT, if the tumor is not completely encircled by the GGO rim, there is a high probability of incomplete treatment.

At the 1-month follow-up, incomplete ablation should be suspected if there is no increase in size of the ablation zone or if the consolidation demonstrates nodular enhancement akin to the original tumor [7,8].

At 6 months, any growth in size of the ablation area is suggestive of recurrence [7].

At all stages of follow-up, the appearance of either central or peripheral nodular or irregular enhancement should be considered as residual or recurrent disease (Figure 4f and Figure 5f), since the ablated area undergoes fibrous transformation, and it should not show contrast enhancement, except for the persisting peripheral safe zone [7,15].

## 5. Early Complications

The most common complications to consider after lung MWA are pneumothorax (38%), pleural effusion (3–6%) and parenchymal hemorrhage (3–6%), which in most cases present a benign course without any consequences for the patient [7,10,14,21,25]. Since these represent frequent findings in clinical practice in most hospitals, we decided to describe a rare complication (0.5%) that often risks being overlooked: bronchopleural fistula (BPF) [25].

**Figure 6:**
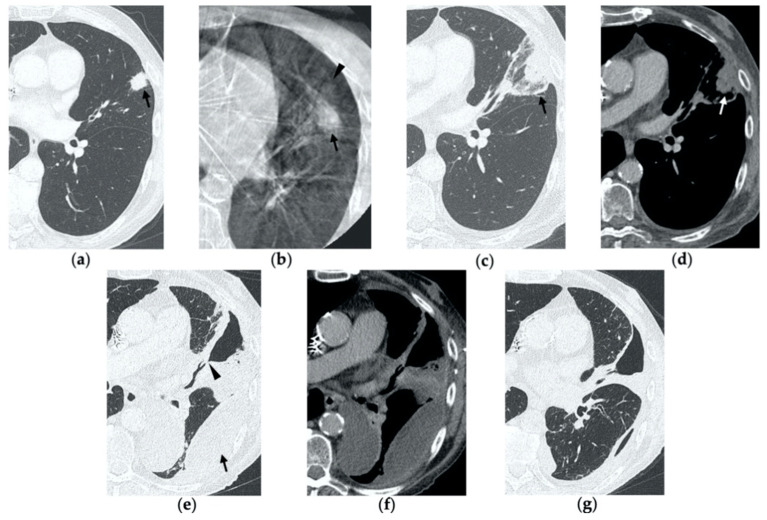
77-year-old man with biopsy proved lung adenocarcinoma in the left upper lobe. (**a**) Axial CT image before treatment (black arrow). (**b**) Cone-beam CT image obtained at the end of the procedure shows GGO (black arrow) encircling the treated lesion and a minimal pneumothorax (arrowhead), which resolved after percutaneous drainage. (**c**,**d**) On axial CT images obtained after 1 month, there is no evidence of pneumothorax and the ablation site shows a large area of increased density, with well-defined margins and a central consolidation with inner cavitation (black arrow), without contrast enhancement (white arrow). (**e**,**f**) Three-month follow-up axial CT images show a large hydropneumothorax (black arrow) near the cavitation that is clearly in communication with a bronchus (arrowhead), consistent with a broncho-pleural fistula. (**g**) Axial CT image of the BPF post-pleural drainage insertion.

**Figure 7:**
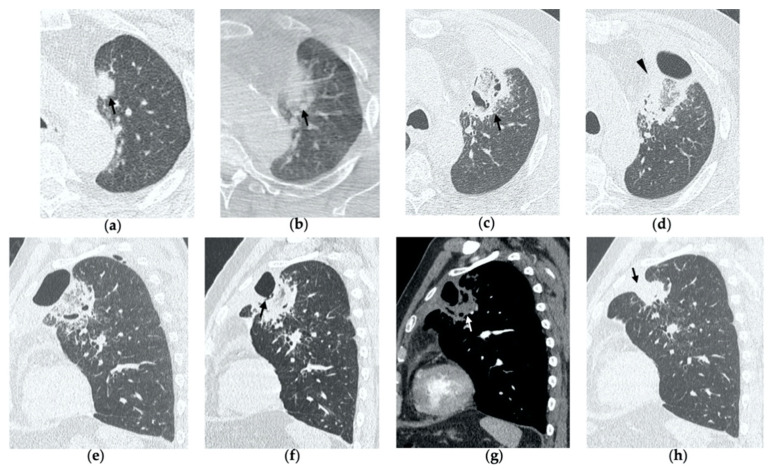
68-year-old man with a biopsy-proved squamocellular carcinoma in the left upper lobe. (**a**) Axial CT before treatment. (**b**) Cone-beam CT image at the end of MWA shows a wide GGO area (black arrow) among the ablation site. (**c**–**e**) Since persistent hydro-pneumothorax on chest X-rays is detected, CT without contrast administration is performed before the usual 1-month follow-up; axial and sagittal CT images show a large consolidation with central cavitation (black arrow); on a different level, a concomitant hydro-pneumothorax is evident (arrowhead); the diagnosis of broncho-pleural fistula is made. (**f**,**g**) Sagittal 2-month follow-up CT images show a decrease in size of the cavitated consolidation and hydropneumothorax; a communication between a bronchus and the pleural cavity is evident (black arrow); (**f**) the consolidation shows no contrast enhancement (white arrow). (**h**) Axial 4-month follow-up CT image shows a decrease in size of the consolidation with complete resolution of both cavitation and pneumothorax (black arrow).

BPF is defined as communication between a bronchus and the pleural space through the ablation zone (Figure 6e,f). The management of this complication is challenging, since treatment may require insertion of a percutaneous drainage or eventually surgery, bronchoscopic or interventional procedures. As a result, BPF represents an extremely rare though potentially severe complication and it should be suspected in cases of delayed or persistent pneumothorax, detected with chest X-ray, respectively, during early and late follow-up CTs (Figure 7) [26].

BPF commonly occurs with hydropneumothorax, and it should not be mistaken with a bronchial fistula, which is a communication between a bronchus and a cavitation in the ablation area and usually resolves uneventfully [26].

## 6. Conclusions

Normal CT appearance, imaging features suggestive of recurrence and the complications that may be misdiagnosed at these stages are summarized in Table 1.

In conclusion, with this pictorial essay, we propose a timing for post-MWA surveillance of lung lesions, with a table of the corresponding expected findings at each scheduled examination, so that non-specialist radiologist may have a ready-to-use guide to interpret imaging.

## Figures and Tables

**Table 1:** CT imaging features.

CT Imaging Features	Post-Procedural CBCT	Contrast-Enhanced CT at 1 Month	Contrast-Enhanced CT at 3 Months	Contrast-Enhanced CT at 6 Months
**Normal**	Well-demarcated oval or wedged-shaped area of hazy GGO, larger than the original lesion	Area or consolidation larger than the original lesion, with hypoattenuating bubbles or a cavity with thin walls, no central contrast enhancement, possible linear rim of hyperenhancement	Decrease in size, resolution of cavitation, possible linear rim of hyperenhancement	Decrease in size; fibrosis scarring
**Suggestive of Recurrence**	Tumor exceeding the area of GGO	No consolidation of the ablation site; nodular enhancement	Increase in size, central or nodular enhancement	Increase in size, central or nodular enhancement
**Complications with Similar Appearance**	**Parenchymal hemorrhage** (poorly demarcated, also along the needle tract)	**Abscess** (cavity with air-fluid level, thick walls and irregular internal contours, clinics of infection) **BPF** (hydropneumothorax with/without evidence of communication between a bronchus and the pleural space through the ablation zone)		

## Data Availability

Not applicable.
